# Carbimazole inhibits TNF-α expression in Fat-induced hypothyroidism

**DOI:** 10.1186/s40200-014-0083-4

**Published:** 2014-08-21

**Authors:** Yamani Bhusan Tripathi, Nidhi Pandey

**Affiliations:** Department of Medicinal Chemistry, Institute of Medical Sciences, Banaras Hindu University, Varanasi, 221005 India

**Keywords:** Obesity, High fat diet, Carbimazole, TNF-α

## Abstract

The effect of the carbimazole on expression of tumor necrosis factor (TNF-α) in liver, was investigated in an experimental model of high fat diet (HFD) induced obesity. The HFD (orally given for 4 months) induced TNF-α in liver tissue along with raised serum triglyceride (TG), cholesterol and high TSH (62%). In carbimazole (1 mg/100 gbw) treatment, the induction of TNF-α was significantly inhibited, without affecting other parameters. It also improved the liver function, which was raised due to HFD in experimental control rats.

## Introduction

The inflammation is the primary cause of metabolic syndrome [MS] [1]. It is associated with faulty life style and food habits [2] and results to ectopic fat deposition [3]. The involvement of adipogenesis and high TSH has been reported [4,5]. The raised inflamation is due to accumulation of M-1 macrophages [6]. Whether this induction is a cause or effect of obesity is debatable. Similar to pre-diabetic patient with impaired glucose tolerance (IGT), where both insulin and glucose levels are higher, [7,8] raised TSH may be adaptive as reported by others also [9]. Here we have investigated role of Carbimazole on high fat diet induced expression of inflammatory markers and tried to correlate the inter relationship between obesity, thyroid function and inflammation.

## Material and methods

The rats were divided into group-1 (maintained normal diet and water). Group −2 [received high fat diet (HFD) (lard [400 g/lit], 20% fructose, casein [80 g/lit], cholesterol] Group- 3 [received HFD + Carbimazole (Abbott, HP, India) (1 mg/100 gbw)]. Above treatments were continued for 4 months and finally rats were sacrificed to assess lipid profile (serum triglyceride (TG) and cholesterol) and liver function test namely aminotransferase (AST), Alanine transaminase (ALT), and alkaline phosphatase (ALKP) and thyroid stimulating hormone (TSH). The expression of TNF-alpha was determined in Liver tissue by RT-PCR [10].

Ethical clearance: The protocol was approved by animal ethics committee of our Institution (IMS, BHU-letter # Dean/2005-06/Animal Ethical Committee/390 dated-18.05.2006).

## Results

**Figure 1:**
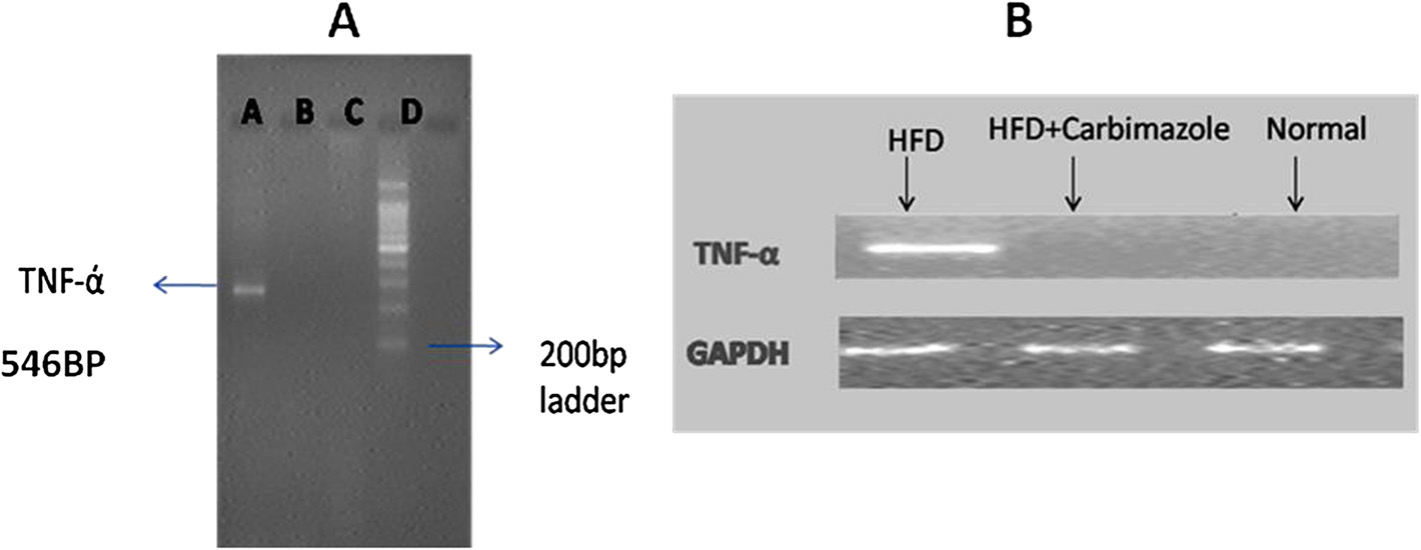
**RT-PCR assessment of TNF-α [4 months] photograph normalized with GAPDH. A**: rats with high fat diet (HFD) **B**: Rats with HFD + Carbimazole, **C**: normal rats, **D**: 200 bp DNA ladder.

Lipid profile, liver function and TSH were significantly raised in animals fed with HFD (Table 1). There was high expression of TNF-α (Figure 1) also in these rats. In Carbimazole treated animals (group 2) there was significant prevention in rise of liver function enzymes and TNF-α, without significant change in serum TG, cholesterol and TSH.

**Table 1:** **Blood biochemistry after 4 months of various treatments**

**Diet type**	**Normal**	**HFD (experimental control)**	**HFD + Carbimazole**
Body weight [g]	165 ± 7.0	162.5 ± 37.5	167 ± 22.1
Triglycerides [mg/dl]	85.8 ± 9.3	115.6 ± 33	124 ± 45
Cholesterol [mg/dl]	72.5 ± 10.2	110 ± 45	96.9 ± 13.6
AST [U/L]	72.5 ± 10.2	126.6 ± 9.9	113.1 ± 12.3*
ALT [U/L]	72.2 ± 10.2	105.9 ± 3.2	92.9 ± 5.73*
ALP [U/L]	441.8 ± 8.2	361.2 ± 30.8	373.5 ± 18.8
TSH [μU/ml]	0.3 ± 0.024	0.8 ± .040	0.7 ± .032

Data presented as mean ± SD. P value: (N = 6), *< 0.05 when compared experimental control with carbimazole treated rat.

## Discussion

It is well documented that faulty diet and life style mediated physiological changes induces systemic low grade inflammation [LGI]. Our results indicate the raised serum TSH and TNF-α in high fat diet fed rats and lower in carbimazole treated rats (when compared to normal control animals of Gr-1). This could be due to inhibitory action of carbimazole on Rac1, involved in expression of TNF-α [11]. Since, TSH level remains same as in experimental control (only HFD rats), there could direct inhibitory effect of TSH on expression of TNF-α as reported earlier in case of osteoclast [12]. High release of Leptin by adipocytes is reported in obesity, which further induces TSH [9]. Accumulation of triglycerides in Gr 2 and Gr3 animals could an adaptive mechanism to reduce circulating free fatty acid, as it is involved in insulin resistance and systemic inflammation. Thus it could be considered as protective mechanism. The raised TSH in obesity further enhances the release of T3 and T4, responsible for rise in thermogenesis and reduction of deposited lipid [13]. Thus low T3 and T4 in established obesity could be an indication of failure of system in counteracting the obesity. Many reports suggest that hypothyroidism could be the cause for obesity. Diez et al. have reported TNF –α mediated destruction of thyroid cells, resulting to low T3/T4 and raised TSH [14]. It is associated with accumulation of adipose-tissue-embedded macrophages in obesity. Higher Leptin in obese, is another factor to increase TSH. Contrary to this, raised TSH inhibits secretion of TNF–α in some cells like osteoclast [12]. This may happen in other tissue also where TSH receptor are reported. Thus, hypothyroid condition in obesity could be an initial step to regulate the abnormal physiology, but it needs further experimental evidences.

## Conclusion

The inhibition of TNF –α expression in carbimazole treated group could be its direct anti-inflammatory effect, but it can also be through high TSH, which needs further exploration.
